# MicroRNA-Mediated Positive Feedback Loop and Optimized Bistable Switch in a Cancer Network Involving miR-17-92

**DOI:** 10.1371/journal.pone.0026302

**Published:** 2011-10-14

**Authors:** Yichen Li, Yumin Li, Hui Zhang, Yong Chen

**Affiliations:** 1 School of Life Sciences, Lanzhou University, Lanzhou, China; 2 Key Laboratory of Digestive System Tumors, Lanzhou, China; 3 Institute of Theoretical Physics, Lanzhou University, Lanzhou, China; John Innes Centre, United Kingdom

## Abstract

MicroRNAs (miRNAs) are small, noncoding RNAs that play an important role in many key biological processes, including development, cell differentiation, the cell cycle and apoptosis, as central post-transcriptional regulators of gene expression. Recent studies have shown that miRNAs can act as oncogenes and tumor suppressors depending on the context. The present work focuses on the physiological significance of miRNAs and their role in regulating the switching behavior. We illustrate an abstract model of the Myc/E2F/miR-17-92 network presented by Aguda et al. (2008), which is composed of coupling between the E2F/Myc positive feedback loops and the E2F/Myc/miR-17-92 negative feedback loop. By systematically analyzing the network in close association with plausible experimental parameters, we show that, in the presence of miRNAs, the system bistability emerges from the system, with a bistable switch and a one-way switch presented by Aguda et al. instead of a single one-way switch. Moreover, the miRNAs can optimize the switching process. The model produces a diverse array of response-signal behaviors in response to various potential regulating scenarios. The model predicts that this transition exists, one from cell death or the cancerous phenotype directly to cell quiescence, due to the existence of miRNAs. It was also found that the network involving miR-17-92 exhibits high noise sensitivity due to a positive feedback loop and also maintains resistance to noise from a negative feedback loop.

## Introduction

MicroRNAs (miRNAs) are small, endogenous non-coding RNA molecules, typically 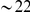 nucleotides (nt) in length. Traditionally, miRNAs were thought to be an undesirable class of small RNAs that only served a relevant function in nonmammalian species. In 1993, Ambros and colleagues found that the lin-4 gene does not encode a protein product, but gives rise to a 61-nt precursor gene that matures to a more abundant 22-nt transcript in the model organism *Caenorhabditis elegans* [1]. The Ruvkun laboratory observed that lin-14 protein synthesis is regulated post-transcriptionally and that lin-14 levels are inversely proportional to those of lin-4 RNA [2]. Thus, they revealed the first miRNA and mRNA target interaction, where the lin-4 RNA has sequence complementarity to the 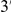 untranslated region of the lin-14 gene. Currently, there have been over 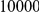 miRNAs identified and published in public databases (miRBase database, http://microrna.sanger.ac.uk). These miRNAs have been identified in animals, plants, and viruses and are involved in the regulation of a variety of biological processes.

By post-transcriptionally down-regulating gene expression, it is currently predicted that miRNAs regulate up to 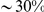 of human genes and their targets include signaling proteins, enzymes, and transcription factors [3]. The diversity and abundance of miRNA targets result in miRNAs playing important roles in nearly all fundamental cellular processes, such as developmental timing, cell proliferation, apoptosis, stem cell maintenance, differentiation, signaling pathways, and pathogenesis including carcinogenesis [4]–[6]. It is remarkable that approximately half of the known miRNAs are located inside or close to fragile sites and in minimal regions of loss of heterozygosity, minimal regions of amplifications, and common breakpoints associated with cancer [7]. Recent studies have shown that miRNAs are involved in the initiation and progression of a variety of cancers and can act as oncogenes or tumor suppressors depending on the tissue and the expression level of their targets [8], [9].

Clearly, miRNAs cannot independently perform a single task in cells. Instead, miRNAs regulate cellular networks as network components in many cellular functions. Indeed, it is thought that miRNAs lead to more effective noise buffering [10], [11]. In addition, Aguda et al. found that miR-17-92 plays an oncogenic role in one setting but suppresses tumor formation in a different scenario [12]. Here, there are two determinative factors, the definition of the postulated cancer zone and the switch behaviors of the system dynamics [12]. Note that the Myc/E2F/miR-17-92 network is composed of two feedback loops: a positive self-feedback loop for protein module (Myc/E2F) and a negative loop between the miRNA and the protein (Figure 2 in [12] or Figure S1). This network appears a typical bistable switch behavior and a one-way switch corresponding to the bistability and monostablility, respectively [12]. In fact, a single positive feedback loop without miRNA is enough to realize a bistable switch. Consequently, it is interesting to investigate the physiological significance of miRNAs or why cancer networks require miRNA regulation and the contributions from miRNAs.

To examine this issue, we focus here on an abstract model of the Myc/E2F/miR-17-92 network described by Aguda et al. [12] and present simulations with experimental parameters. Our results show that the existence of miRNA improves the ability of the bistable switches in the systems. For the single-loop switch, there is a so-called fast/slow loop to describe the fast/slow response kinetics for the activation and inactivation [13]. Normally, the single fast-loop switch achieves more rapid responses. Moreover, we also found that miRNA can mediate the system between a fast loop and a slow loop due to the different rate coefficients of degradation for miR-17-92 and E2Fs/Myc (in general, miRNAs is more stable than protein), and can diversify the response behavior of the system to the input stimulus. Especially, the undamped relaxation oscillation behavior of the system indicates a possible digital regulation mode. Furthermore, the switching behaviors of the network involving miR-17-92 is both sensitive to stimuli and resistant to fluctuations in stimulus. Our finding show that miRNAs play a key role in the possibility to achieve a sensitive robustness in biological systems.

## Results

### Model Formulation

**Figure 1:**
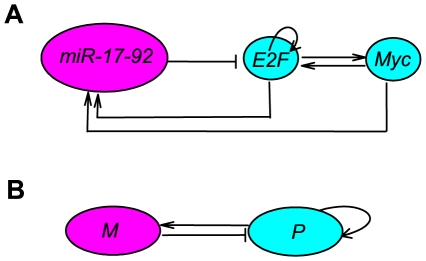
Schematic illustration of the cancer network involving miR-17-92, E2F, and Myc. 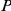
 and 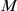 denote the protein module (Myc and E2Fs) and the miRNA cluster, respectively.

By following the mammalian G1-S regulatory network, the essential abstract structure of the Myc/E2F/miR-17-92 network is illustrated in Figure 1. 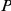 denotes the protein module (Myc and E2Fs), and 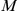 is the miRNA cluster module (see Ref. [12] or Figure S1 for the detailed reduction process). The positive feedback loop in module 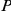 represents an autocatalytic process, which is also inhibited by module 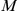. At the same time, 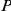 induces the transcription of 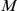.

The dynamics of the respective concentrations of 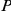 and 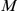 are described by the following ordinary differential equations [12]:
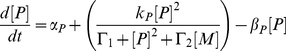
(1)

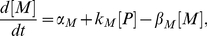
(2)where 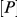 and 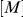 denote the concentrations of 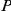 and 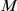, respectively. 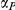 describes the constitutive protein expression from the signal transduction pathway in the extracellular medium and is experimentally controlled in the cell culture medium. 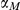 depicts the 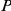-independent constitutive transcription of 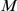. 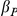 and 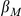 are the rate coefficients of degradation. 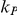 is the constant of protein expression, and 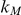 is the rate constant. 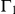 is the coefficient of protein expression, and 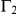 is a measure of the miRNA inhibition of protein expression.

Under a series of nondimensionalizing processes [12], Eqs. (1) and (2) can be rewritten as:
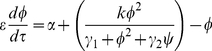
(3)

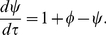
(4)Here, 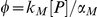, 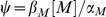, and 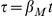. In general, 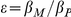 is less than 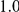 because miRNA is more stable than protein. 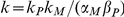 is allowed to vary in the range of 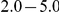. The experimentally controllable parameters 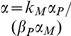 and 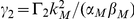 vary from 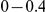 and 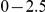, respectively [12], [14], [15]. The last parameter 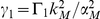 is set as 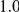. The process to deduce the dimensionless parameters is presented in Text S1.

### Steady States, One-way Switches, and bistable Switches

Setting Eqs. (3) and (4) equal to zero and solving for the roots of the algebraic equations from the right-hand sides, we obtain the steady states of the model. As shown in [12], there exists the following relationship:
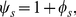
(5)where 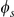 and 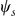 represent the steady states of 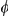 and 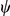, respectively (see the explicit solutions in Text S2). Note that there is only a constant difference between 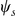 and 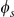. Moreover, 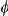 denotes the dimensionless concentration of protein E2Fs and Myc which is directly correlated with the oncogene or tumor suppression. Thus, we present only the results of 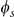 and 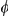 in the following context.

**Figure 2:**
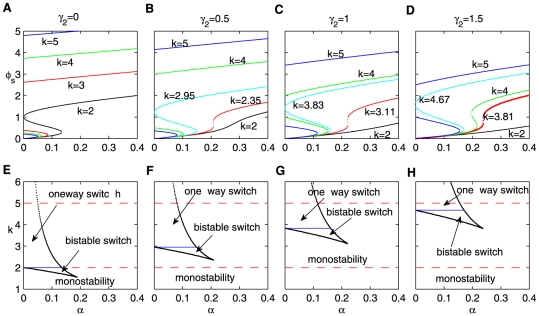
Steady-state bifurcation diagrams of the dimensionless protein concentration 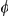
 **(top panels) and phase diagrams (bottom panels) of switching behavior.** The strength of the dimensionless measure of miRNA inhibition 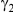 is increased from 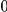 to 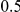, 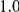 and 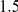 (from left column to right one). In the bottom panels, the red dashed lines denote the range of the protein expression constant 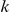, from 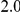 to 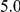. Clearly, the system is greatly improved with regard to the ability of the toggle switch with the inclusion of miRNA inhibition 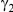. Here, 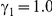.

Due to the positive feedback in module 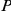, this system exhibits a switching behavior, and it has two stable fixed points in the appropriate parameter regime [16]. Steady-state 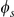 bifurcation diagrams as a function of the parameter 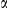 for different values of 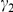 are presented in the top panels of Figure 2. Considering the physiological constraints, the horizontal axis 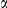 should be greater than 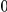 and terminates at a maximal value of 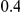. Clearly, it also shows the oncogenic and tumor suppressor properties of miR-17-92 using the concept of the cancer zone [12] (see Figure 2A–D). Note that 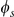 decreases with decreasing 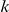 for fixed 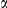 and 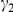. Therefore, the positive feedback loop has a similar reversely regulating function of miRNA.

In the case of a bistable switch, the system exhibits hysteresis, which is a property of bistable systems. For convenience, we denote the lower/upper steady state (lower/higher protein concentration) as the off/on state. As an example, for 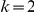 in Figure 2A, as 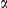 increases along the lower stable branch, 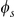 remains in the off-state until 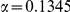. When 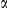 is increased further, the off-state vanishes, and the switch moves towards the upper stable branch, corresponding to the on-state. Then, if we decrease 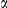, the switch moves along the upper stable branch until 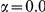, and then a transition brings the switch back to the off-state. However, the system also acts as an extreme manifestation of hysteresis, a one-way switch, where the on-state branches into the negative domain but is actually eliminated due to a physically meaningful restriction (e.g., 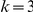, 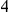, 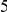 in Figure 2A and 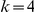, 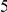 in Figure 2B).

It is well-known a bistable region is enclosed by two saddle-node bifurcation points (the left and right knees of the curves in the top panels of Figure 2). For any 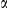 values in this region, the system has two stable solutions and one unstable one. The bottom panels of Figure 2 provide whole phase diagrams of switch behaviors including bistable and one-way switches and a monostable region for different 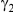 values. By increasing the strength of the positive feedback 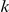, the system undergoes a transition from monostability to a bistable switch and then to a one-way switch. The parameter 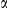 exhibits a similar influence. When we increase 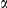, the transition begins with a one-way switch to a bistable switch and then to monostablility. As for the dimensionless parameter of miRNA inhibition 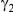, it reduces the switch region enclosed in the effective domain of 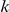 (from 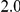 to 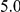, denoted by two red dashed lines). However, it should be noted that the system only has one-way switching behavior without 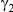 (see Figure 2E). The effective region of the bistable switch is expanded by increasing the strength of 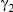 (Figures 2E–H). Otherwise, for higher values of 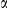, the system is always monostable, but in the case of lower 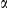 values, the system produces diverse dynamical behaviors with the existence of 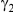.

**Figure 3:**
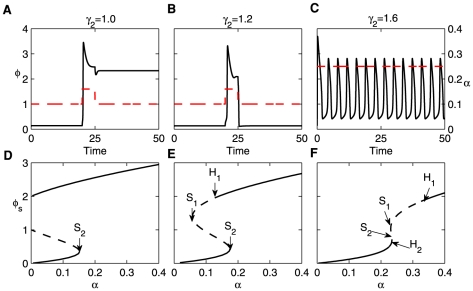
The dynamical behaviors of the system when increasing the inhibition of miRNA 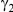
 **.** The strength of positive feedback is set at 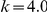, and the strength of negative feedback 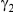 is increased from 1.0 to 1.2 and 1.6 from left to right. Parameter 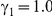 and 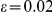. The upper rows show the time course of the 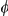 response to (A and B) the pulse input, 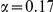 for 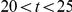 and 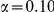 for others, or (C) a constant stimulus with 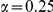, where the red dashed lines denote the input signal. The lower rows show the corresponding bifurcation diagram, where 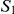/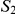 denotes saddle point and 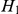/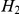 represents a Hopf bifurcation. Clearly, the system undergoes transitions from bistability to excitability and to relaxation oscillation with increasing 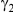.

Note that there exists a cancer zone between levels associated with normal cell cycles and apoptosis, a range of Myc and E2F levels with increased probability of inducing cancer (see Figure S2 or Figures 3, 6 in [12]). In general, the off-state denotes cell quiescence and the on-states represent other processes (cell cycles, the cancer zone, and cell apoptosis). In all cases, the regulation imposed by miRNA or the positive feedback loop is confined to the upper state, from cell cycles to the cancer zone and to cell apoptosis or the inverse order (see Figure 2 and Figure 6 in [12]). Considering a perturbation of 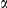 along the on-state of a single bistable switch, 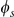 changes slightly. For 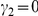, the system almost stays in the on-state because there only exists the one-way switch (In fact, the probability of transferring to another cell status is also very low. See Figure 2A.) however, for 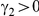 (the existence of miRNA), it is possible to return to cell quiescence due to the bistable switch (Figures 2B–D). This means that the miRNAs lead to the recurrent process from the on-state to the off-state. Thus, the miRNA provides two possible regulation pathways, from cell apoptosis or the cancer zone to cell quiescence (Figure S3).

### Diversification of the Signal-response Behaviors

As shown in Figure 2, the emergence of the inhibition of miRNA 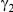 induces diversification of the system dynamics in the physiological regions of 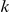. In fact, the repression of 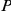 by 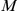 yields negative feedback (see Figure 1). This system, composed of positive and negative feedback, is a flexible motif that can exhibit various behaviors [17], [18]. It has already been shown that the negative feedback makes oscillation possible [19], [20]. Here, we show that the system can exhibit diverse signal-response behaviors corresponding to the different regimes of Figures 2F–H.

For any fixed strength of the positive feedback 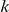, the behavior of the system is different for varying 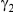 (see the bottom panels of Figure 2). With 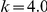 and 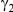 ranging from 1.0 to 1.2 and 1.6, Figure 3 shows that the response of the system to the input signal is tuned by 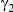. The top row shows the time evolution of 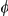 under the input stimulus, and the bottom row depicts the corresponding bifurcation diagram of the system. The solid and dashed lines denote stable and unstable steady states, respectively. 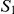/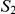 denotes a saddle-node bifurcation and 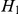/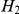 represents a Hopf bifurcation. The input stimulus is a pulse with 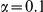 except for when it is 0.16 from time 20 to 25 in Figures 3A and 3B, but it always is constant at 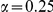 in Figure 3C. The initial values used were 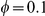 and 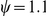.

First, in the case of 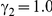, the system behaves irreversibly. Under the pulse input, the system settles to the on-state and cannot return to the initial off-state (Figure 3A). This behavior is typical of a one-way switch (Figure 3D). As 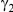 is increased to 1.2, the strength of the pulse input is in the range between 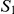 and 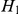 (Figure 3E). In this case, there are three steady states, but only the lower state (off-state) is stable while the others (the upper and middle ones) are unstable. The system exhibits excitability where 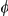 is first driven to the on-state due to the instability from the pulse input and then completely recovers to the original off-state after a short shift (Figure 3B). Thus, the model system also generates a large-amplitude transient pulse to respond to the pulse input.

When 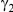 is further increased and the strength of the constant stimulus is between two Hopf bifurcation points 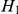 and 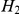, the system enters into limit-cycle oscillations (see Figures 3C and 3F, and the respective 3-dimension bifurcation in Figure S4). 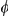 rises gradually and then drops rapidly after reaching a maximum, and the cycle repeats. As we all know, the undamped relaxation oscillation is a periodic process in which slow smooth change of the state of an object over a finite interval of time is alternated with rapid irregular change of the state during an infinitely short time. Since van der Pol presented the classic example of a one-dimensional system having relaxation oscillations [21], such oscillatory processes are observed in many real mechanical, radiotechnical, biological, laser physical etc., objects. So, such an oscillation is a type of undamped relaxation oscillation. Notably there is no time delay in the recent limit-cycle oscillation system. This means that this oscillation resulted from the hysteresis induced by the positive feedback [22], [23].

As stated in Figure 3A and 3D, the system exhibits a fundamental phenomenon in nature, the so-called bistability. A bistable system is able to rest in two states, which need not be symmetric. The defining characteristic of bistability is simply that two stable states (minima) are separated by a barrier (local maximum). For example, for an ensemble of particles, the bistability comes from that free energy has three critical points. Two of them are minima and the last is a maximum. However, Figure 3B and 3E display the excitability of the system. Common to all excitable systems is the existence of a rest state, an excited (or firing) state, and a refractory (or recovery) state, such as action potential in neural systems. The system is in the presence of one stable and one or more unstable fixed points. If unperturbed, the system resides in the rest state; small perturbations result only in a small-amplitude response of the system. For a sufficiently strong stimulus (for example, larger than the 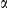 value of 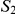 in Figure 3E), the system can leave the rest state, going through the firing and refractory states and then comes back to rest again [24].

**Figure 4:**
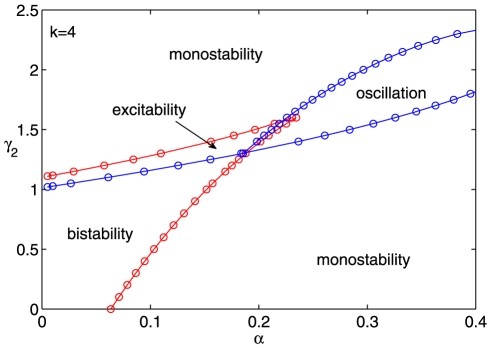
Bifurcation diagram in the space spanned by 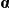
 **and** 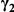 **with** 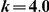 **,** 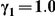 **.** The bulk diagram of the dynamical behavior of the system is composed of four regions: monostability, bistability, excitability, and undamped relaxation oscillation. The red and blue circles on borderlines denote the saddle-node and Hopf bifurcations, respectively.

As illustrated above, with increasing 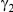, the system undergoes a transition from bistability to excitability and to undamped relaxation oscillation without a time delay. We suggest that there is a feasible way to produce diverse signal-response behaviors by combining the inhibition of miRNA 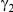 and the experimentally tunable parameter 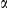. Figure 4 presents an overview of the tunability using the phase diagram of the system dynamics in 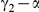 plane with 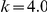. The bulk diagram is composed of four kinds of dynamics: monostability, bistability, excitability, and undamped relaxation oscillation where the borderlines between these dynamics (solid lines in Figure 4) are saddle-node and Hopf bifurcation points, respectively. We cannot observe the codimension 2 or other higher codimension bifurcations in our studied parameter range. It is obvious that the values of 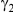 and 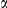 can be cooperatively tuned in the corresponding regions to achieve desirable behaviors and functions. In fact, when we fix any one parameter in 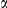, 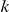, and 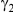, the others can perform similar synergetic function to achieve the diverse response behaviors (see Figure S5).

Indeed, a single positive feedback loop with ultrasensitivity is able to act as a bistable switch and a single negative feedback loop with a time delay can produce sustained oscillations [25]–[27]. However a motif assembled by a positive loop and a negative loop not only performs the both functions without changing the topological structure, but also presents excitable behavior. Note that the last two behaviors resulted from the negative feedback loop of miR-17-92 because there can only be one-way switches without miRNAs (Figure 2A). These signal-response behaviors offer diverse regulating options. The oscillation (pulses) could provide potential precise regulation, such as the digital response of p53 to DNA damage [28], [29].

### Optimized Bistable Switch

According to Eqs. (3–4), the dimensionless parameter 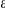 is a time constant for the activation and inactivation of 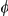 and determines whether the switch is fast or slow. Note that 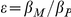 is the ratio of the degradation rate for E2Fs and Myc for miR-17-92. Consequently, the switching behavior of the protein concentration from the positive feedback loop can also be represented as interlinked dual-time feedback loops [13], [30], [31].

**Figure 5:**
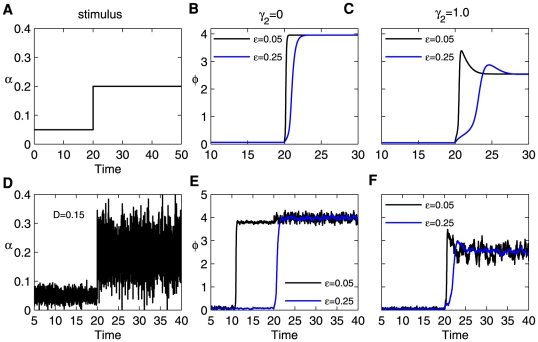
Responses of switches to a step stimulus input. (A) The jumping stimulus input from 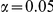 to 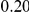 at time 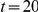. (B) The responses of the system for a fast loop 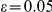 (red) and a slow loop 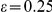 (blue), where 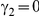. (C) The same as (B) but with 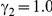. (D) The same as (A) but with an imposed fluctuation 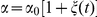, where 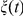 is Gaussian white noise with variance 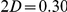 and mean 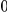, and 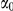 is the same as in (A). (E–F) The same as (B) and (C), respectively. All simulations used 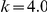 and 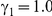.

Figure 5 illustrates the switch responses of the system for two different time constants 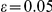 and 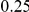 to a step stimulus with or without fluctuation. The basal strength of the stimulus strength is set at 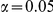, where the system is initially in the off-state, and then jumps to 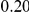 at time 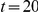, corresponding to the on-state of the system. The panels in the left column denote the stimulus input, where the left bottom panel corresponds to a fluctuating environment described by Gaussian white noise with mean 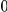 and variance 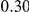 (Figures 5A, 5D). The middle and right columns show the response of the switch for 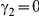 and 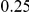, respectively. In all of the cases, the system with a smaller 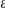 increases rapidly at the initial stage and responds much faster than that of the slower loop. The fluctuation amplitude of 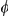 for larger 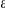 values is much smaller than that of the faster loop (Figures 5E, 5F). That is, the fast loop is critical for the switching sensitivity, but the slow loop increases the switching stability to resist stimulus fluctuations. Furthermore, the inhibition of miRNA 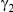 also slows down the switching process, especially near the on-state, and effectively represses fluctuations (Figures 5B and 5C, Figures 5E and 5F).

To clearly investigate the switching robustness to fluctuations in the stimulus, we use the definition of the fraction of transition 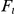 [17], [30]. The system is driven by the same noisy stimulus 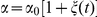 with different seeds, where 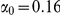 and 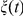 is Gaussian white noise. Initially, we settled on a population of 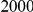 cells in the on-state of the system, with some cells that may flip to the off-state due to a noise-induced switch. 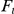 is defined as the ratio of the number of cells in the transition state to the number of all cells at each time point. Moreover, one can define the time required for 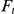 to reach the midpoint between its initial and steady-state values as the so-called response time 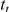.

**Figure 6:**
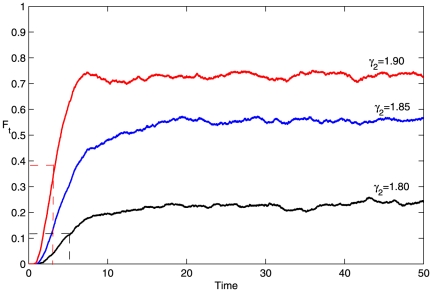
Time course of fluctuation-induced escape from the on-state (upper state) to the off-state (lower state). Each time course represents the evolution of the fraction 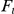 that has transitioned at least once to the off-state, for an ensemble of 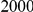 cells. Here, the parameters are 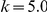, 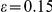, 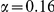, 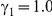, and a Gaussian white noise with variance 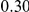.

Figure 6 displays the time courses of 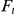 for 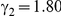, 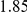, and 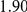. For smaller 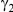 values, it takes a relatively long time for 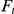 to reach a smaller steady state. We denote 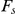 as the value of the steady state of 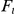. For example, 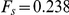 and 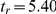 for 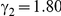, which means that most of cells are still trapped in the on-state for a long time. In the case of 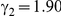, 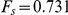 and 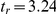 indicating that a significant number of cells (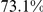) have flipped to the off-state and are taking less time to reach the steady state of 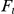.

**Figure 7:**
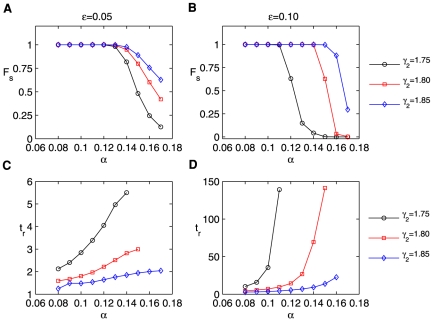
Noise tolerance and response features of the systems including the inhibition of miRNA. (A) Steady values of the fraction 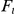 of transition from the initial on-state to the off-state as a function of 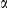 for 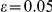. (B) The same as (A) for 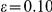. (C–D) The response time 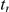 *vs* 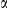 for 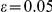 and 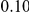, respectively. All of the simulations used 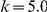, 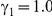, and a Gaussian white noise with variance 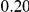.

Furthermore, we show the fraction of transition 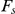 and the response time 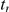 as functions of 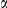 for different 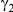 and 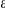 values in Figure 7. In the simulations, all 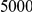 cells initially settled in the on-state. The fluctuation of 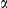 follows a Gaussian white noise distribution with variance 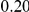. With increasing 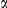, 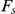 decreases from 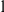 to 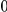, but 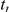 also increases quickly in the case of 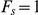. Especially in the flipping region of 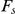, 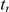 decreases when 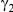 increased, as can be similarly observed in Figure 6. These switching behaviors resulted from the binding between the on-state and the off-state decreasing when 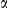 or 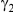 increases in the region of the bistable switch (see the dynamic diagram of 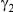 *vs* 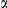, Figure S6).

Note that 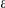 is 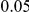 in the left column of Figure 7 and is 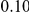 in the right one. Obviously, for smaller values of 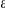 (fast loop) and larger values of 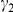, there exists a larger region of 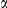 for which almost all cells are trapped in the on-state (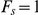). In the critical region of 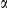 for 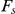 from 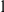 to 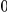, the flipping process with small 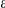 values is less sensitive to 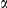 than with larger ones (Figures 7A and 7B). In addition, 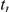 for small 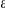 values is significantly less than for larger ones (Figures 7C and 7D).

Cellular processes are essentially stochastic and occur in a fluctuating environment [32]. A small perturbation in the stimulus input could be amplified by positive feedback [33], [34]. (Sometimes, positive feedback can work as a noise-filtering device [35]). Additionally, noise-induced switching behavior may induce a false decision regarding cell fate (case 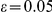 in Figure 5E). By involving the negative feedback loop of miRNAs, the Myc/E2F/miR-17-92 cancer network operates like a dual-time switch interlinked by fast and slow positive loops. The entire system exhibits high noise sensitivity in the off-state due to the rapid responses of the positive loop, which is regulated by the ratio between the miRNA and protein degradation rates (note that 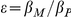 is often much less than 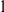). At the same time, the system is resistant to noise when it is in the on-state as a result of the negative feedback.

## Discussion

It has been reported that miR-17-92 behaves as an oncogene and a tumor suppressor depending on different situations [36], [37]. For the first time, Aguda et al. analyzed the reduced model of the coupling between the E2F/Myc positive feedback loops and the E2F/Myc/miR-17-92 negative feedback loop. They showed that miR-17-92 plays a critical role in regulating the protein levels (on/off). Most important, they demonstrated the parallel oncogenic and tumor suppressor properties of miR-17-92 using the concept, cancer zone (Figure S2 or Figure 3 in [12]). By considering the bistable switch behaviors, Aguda et al. [12] predicted that increasing miRNA level drives E2F/Myc level in normal cell cycle to enter the cancer zone (oncogene, case a), or drives protein levels to exit cancer zone and enter the cell apoptosis (tumor suppressor, case b), and vice versa (see Figure S2 or Figure 3 in [12]). The reduced abstract model of the Myc/E2F/miR-17-92 network is typically interlinked by positive and negative feedback loops (Figure 1). A bistable system with interlinked loops has been illustrated in the yeast galactose-utilization network [38], [39], the mitogen-activated protein kinase 1,2/protein kinase C signaling network [40], [41], circadian clocks [42], [43], the eukaryotic cell cycle [44], [45], the p53-Mdm2 network [29], and so on. It has been shown that a system with interlinked loops behaves as a tunable motif and performs diverse behaviors [17]. The essential dynamic in the Myc/E2F/miR-17-92 network is a bistable switch, which can be realized only by a positive feedback loop without miRNAs (Figure 2A). Thus, the physiological importance of miRNAs remains unclear. The present work is based on the hypothesis that the miRNAs are essential in optimizing the switching behavior of the Myc/E2F/miR-17-92 network, and we focus on the role of miR-17-92 on the response-signal behavior without or with noise.

In this paper, using simulation parameters that are biologically plausible, we have shown that the system represents various behaviors (monostability, a bistable switch, a one-way switch) instead of a simple one-way switch because of the existence of miR-17-92 (see Figures 2E–H. The bistable region is also larger with miRNA present). As a result, the system is capable of generating a diverse array of signal-response behaviors with suitable combined parameters (Figures 3A–C). Especially, we find that, due to the the existence of miR-17-92, the range (parameter 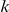) of normal cell cycles is enlarged and this transition (from cell death/cancer to quiescence) is probably realized by noise-induced switches. In addition, the response time constant of the protein module can be regulated by the miRNA degradation rate (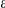 in Figures 5B–C). The Myc/E2F/miR-17-92 network can run as a dual-time switch (interlinked fast and slow positive loops) and appears to be more sensitive to stimuli and resistant to stimulus fluctuations (Figures 5E–F and Figure 6). It means that miR-17-92 can perform efforts to optimize the bistable switch where miR-17-92 confers signaling robustness (to limit undesired signaling fluctuations, buffering effect) and achieves optimal signaling efficacy (balancing effect).

In addition, processes in gene regulatory systems are typically subject to considerable delays induced by underlying biochemical reactions. Time delays in combination with a positive/negative feedback loop can induce sustained oscillations and multistability [12]. The model of the Myc/E2F/miR-17-92 network should also account for multiple time delays. It has been shown that the inclusion of a long-time delay in a negative feedback loop can generate oscillations and that the addition of a positive feedback loop can increase the oscillation amplitude and widen the stimulus regime for the oscillation, thus promoting the robustness of the oscillations [17], [46]. Therefore, it is conjectured that the effect of a time delay would not qualitatively change our results.

Because an individual miRNA usually targets many genes that are involved in various cellular signaling pathways [11], modulations in the level of a single miRNA could eventually affect many pathways at the same time. The miR-17-92 cluster, which comprises six miRNAs (miR-17, miR-18a, miR-19a, miR-20a, miR-19b-1, and miR-92-1), plays an important role in the cell cycle (by targeting the E2F family) [47], apoptosis (by downmodulating the antiapoptotic protein Bim and tumor suppressors PTEN and p21) [48]–[50], and angiogenesis (activated by c-Myc and VEGF) [51]. In this study, the model is constrained in a network associated with the cell cycle, without considering other networks. It would be worthwhile to construct a large-scale gene regulation network including different biological functions of a small group of miRNAs and to develop potential strategies of miRNA-based therapeutic targets.

## Materials and Methods

All numerical bifurcation analyses of the ordinary differential equations were performed with OSCILL 8.28 [52]. The ordinary differential equations and stochastic differential equations were numerically solved and separately integrated using the fourth-order Runge-Kutta scheme and the fourth-order stochastic Runge-Kutta scheme [53], [54] in Fortran 95 codes, respectively.

## Supporting Information

Text S1 **The deduction of the dimensionless parameter ranges.**
(PDF)

Text S2 **The explicit solutions for the steady states of protein and miRNA levels.**
(PDF)

Figure S1
**Reducing process of the Myc/E2F/miR-17-92 network to an abstract model.** Solid arrows mean activation and hammerhead means inhibition. (A) Summary of the interactions among the transcription factors Myc, E2F, and miR-17-92 cluster. (B) The first step in the model reduction. (C) The final abstract model is composed of the protein module 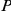 (Myc/E2F) and the miRNA module 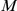 (miR-17-92). It is also presented in detail in Figures 1 and 2 by Aguda et al. [12].(EPS)

Figure S2 **The schematic diagram of cancer zone.** Clearly, the miR-17-92 clusters act as an oncogene or as a tumor suppressor designed by Aguda et al. [12].
(EPS)

Figure S3
**The switching behavior between cell statuses corresponding to the transcriptional activities of E2F or Myc.** Note that the plausible experimental range of 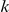 is 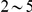. Without the inhibition of miRNAs (left panel), the switch is limited in on-states (cell cycle, cancer, and cell death). Moreover, most of cells are settled on cancer or apoptosis which return back to cell cycles only by decreasing the positive feedback 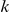. For example, the black dashed-line arrows from 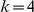 to 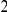 denotes a regulation from cell death to cell cycles. In the presence of miRNAs (right panel), the protein concentration of steady states 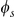 is significantly less than that in left panel. So, the range of normal cell cycles is enlarged and the regulation of 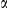 is also at work effectively. Especially, it is possible that cells back to quiescence from cancer or apoptosis directly. As an example, for 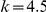, there exists noise-induced switch from cancer zone to cell quiescence (the red dashed-line arrow). Similarly, the regulation of 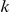 can also realize the same function (see the black dashed-line arrow, regulation from 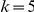 to one optional value between 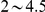). Here parameter 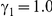.(EPS)

Figure S4
**The 2-dimension phase portraits for** 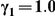 **and** 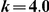 **.** The systems come into steady states for 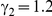 and (A) 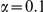, (C) 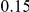. In the case of 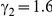, the systems appear periodic oscillations for (B) 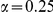 and (D) 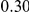. The circles and the solid square denote the initial status (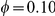, 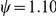) and the fixed steady states, respectively. The arrows indicate the time evolution of the systems.(EPS)

Figure S5
**The diverse signal-response behaviors tuned by the positive feedback** 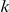 **and the miRNA inhibition** 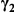 **for fixed** 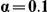 **.** Note that the oscillation region appears for the high 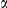 (normally, 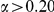), but the bistable switch disappears. Parameter 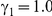.(EPS)

Figure S6
**The bifurcation diagram spanned by** 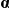 **and** 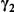 **for** 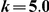 **and** 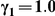 **.** Obviously, in the region of the bistable switch, the range of 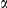 or 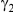 between on-state and off-state decreases with increasing 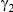 (panel A) or 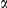 (panel B), respectively. The additional red dashed lines are the drawing borderlines between bistable and one-way switches under different preconditions (for a fixed value of 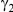/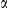 in panel A/B).(EPS)
