# Supplementary material for: MicroRNA-Mediated Positive Feedback Loop and Optimized Bistable Switch in a Cancer Network Involving miR-17-92
Source: PLoS One. 2011 Oct 14;6(10):e26302. doi: 10.1371/journal.pone.0026302 (PMC3194799; doi:10.1371/journal.pone.0026302)
Supplement: Text S1 — The deduction of the dimensionless parameter ranges. (PDF) [file pone.0026302.s001.pdf]

(Li *etc* – *MicroRNA-mediated Positive Feedback Loop and Optimized Bistable Switch in a Cancer Network Involving miR-17-92.)*

1 Deduction of the dimensionless parameter ranges

The reducing process of the dimensionless equations (3) and (4) is the same as in Aguda et al. Moreover, the parameter values in Eqs. (3-4) are also presented by Aguda et al. (see page 19680 in [1]).

The dynamical equations for P and M are

$$\frac{d[P]}{dt} = \alpha_P + \left(\frac{k_P[P]^2}{\Gamma_1 + [P]^2 + \Gamma_2[M]}\right) - \beta_P[P] \tag{1}$$

$$\frac{d[M]}{dt} = \alpha_M + k_M[P] - \beta_M[M]. \tag{2}$$

Considering a similar reducing process [1],

$$\phi = \frac{k_M}{\alpha_M}[P] \quad \psi = \tfrac{\beta_M}{\alpha_M}[M] \quad \tau = \beta_M t$$
$$\varepsilon = \frac{\beta_M}{\beta_P} \quad \alpha = \tfrac{k_M}{\beta_P\alpha_M}\alpha_P \quad k = \frac{k_P k_M}{\alpha_M\beta_P}$$
$$\gamma_1 = \frac{k_M^2}{\alpha_M^2}\Gamma_1 \quad \gamma_2 = \tfrac{k_M^2}{\alpha_M\beta_M}\Gamma_2.$$

Note that, typically, β_P for E2F1 and Myc are $\approx 0.25\,\mathrm{h}^{-1}$ and $\approx 0.7\,\mathrm{h}^{-1}$ from [2], respectively. So, the parameter for whole protein module β_P is expected as $0.25 \sim 0.7\,\mathrm{h}^{-1}$. Similarly, $\beta_M \approx 0.02\,\mathrm{h}^{-1}$, $k_P \approx 0.4\,\mu M\mathrm{h}^{-1}$, and $\Gamma_1 \approx 0.1\mu M^2$ [1–3]. Now, we set $k_M/\alpha_M \approx 3\mu M^{-1}$ and then

$$\gamma_1 = \left(\frac{k_M}{\alpha_M}\right)^2\Gamma_1 \approx 3^2 \times 0.1 = 0.9 \approx 1.0 \tag{3}$$

$$k = \frac{k_P k_M}{\alpha_M\beta_P} \approx \frac{3 \times 0.4}{\beta_P} \Rightarrow k \in (1.71, 4.8) \Rightarrow \text{Approximately,} \quad k \in (2.0, 5.0). \tag{4}$$

In addition, α_P is from 0 to 0.1 $\mu M\mathrm{h}^{-1}$ [1]. So, the limit value of α is

$$\alpha_{max} = \frac{k_M}{\beta_P\alpha_M} \approx \frac{3.0 \times 0.1}{\beta_P} \Rightarrow \alpha_{max} \in (0.43, 1.2). \Rightarrow \alpha_{max} \approx 0.4.$$

Note that α denotes the whole protin module. So, the minimal value 0.43 is the realizable maxmal vale. As a result, it should be

$$\alpha_{max} = 0.43 \approx 0.4 \Rightarrow \alpha \in (0, 0.4). \tag{5}$$

As for γ_2, experimentally, $k_M \approx 0.4\mu M\mathrm{h}^{-1}$, $\Gamma_2 \approx 0.03\,\mathrm{h}^{-1}$ [2]. So,

$$\gamma_2 = \frac{k_M^2}{\alpha_M\beta_M}\Gamma_2 \approx \frac{3}{0.02} \times 0.4 \times 0.03 = 1.8. \tag{6}$$

Here $\gamma_2 = 1.8$ is not the limit value. Considering the limitation to do experiments in whole ranges of all parameters, we set $\gamma_2 \in (0, 2.5)$ as in [1]. Indeed, it is enough to study the dynamics of the system. For $\gamma_2 > 2.5$, the system almost appears a unmeaning monostablility, e.g. Figure 4 in our manuscript.

References

1. Aguda BD, Kim Y (2008) MicroRNA regulation of a cancer network: Consequences of the feedback loops involving miR-17-92, E2F, and Myc. Proc Natl Acad Sci USA 105: 19678-19683.

2. Yao G, Lee TJ, Mori S, Nevins JR, You L (2008) A bistable Rb-E2F switch underlies the restriction point. Nat Cell Biol 10: 476-482.

3. Khanin R, Vinciotti V (2008) Computational modeling of post-transcriptional gene regulation by microRNAs. J Comput Biol 15: 305-316.
