# Supplementary material for: MicroRNA-Mediated Positive Feedback Loop and Optimized Bistable Switch in a Cancer Network Involving miR-17-92
Source: PLoS One. 2011 Oct 14;6(10):e26302. doi: 10.1371/journal.pone.0026302 (PMC3194799; doi:10.1371/journal.pone.0026302)
Supplement: Text S2 — The explicit solutions for the steady states of protein and miRNA levels. (PDF) [file pone.0026302.s002.pdf]

(Li *etc* – *MicroRNA-mediated Positive Feedback Loop and Optimized Bistable Switch in a Cancer Network Involving miR-17-92.)*

1 The explicit solution for the steady states

The steady states of the system leads to

$$\begin{aligned} \alpha + \left(\frac{k\phi_s^2}{\gamma_1 + \phi_s^2 + \gamma_2\psi_s}\right) - \phi_s &= 0 \\ 1 + \phi_s - \psi_s &= 0. \end{aligned}$$

So, we obtain the following the cubic polynomial,

$$\phi_s^3 + c_2\phi_s^2 + c_1\phi_s^1 + c_0 = 0, \tag{1}$$

where

$$\begin{aligned} c_2 &= \gamma_2 - \alpha - k \\ c_1 &= \gamma_1 + \gamma_2(1-\alpha) \\ c_0 &= -(\gamma_1 + \gamma_2)\alpha. \end{aligned}$$

The solutions of a cubic function can be found in many textbooks [1–3]. Define q and r as

$$q = \frac{1}{3}c_1 - \frac{1}{9}c_2^2, \qquad r = \frac{1}{3}(c_1c_2 - 3c_0) - \frac{1}{27}c_2^3,$$

and set

$$\begin{aligned} z_1 &= \sqrt[3]{r + (q^3 + r^2)^{1/2}} \\ z_2 &= \sqrt[3]{r - (q^3 + r^2)^{1/2}}. \end{aligned}$$

So, we obtain the roots of cubics,

$$\phi_{s1} = (z_1 + z_2) - \frac{1}{3}c_2 \tag{2}$$

$$\phi_{s2} = -\frac{1}{2}(z_1 + z_2) - \frac{1}{3}c_2 + \frac{i\sqrt{3}}{2}(z_1 - z_2) \tag{3}$$

$$\phi_{s2} = -\frac{1}{2}(z_1 + z_2) - \frac{1}{3}c_2 - \frac{i\sqrt{3}}{2}(z_1 - z_2). \tag{4}$$

Here, if

$$\begin{aligned} q^3 + r^2 &> 0 \quad \text{one real root and two complex conjugate roots} \\ q^3 + r^2 &= 0 \quad \text{all roots are real and at least one multiple root} \\ q^3 + r^2 &< 0 \quad \text{three distinct real roots.} \end{aligned}$$

References

1. http://en.wikipedia.org/wiki/Cubic_function#Roots_of_a_cubic_function
2. Irving, RS (2004) *Integers, polynomials, and rings*. Springer-Verlag New York, Chapter 10 ex 10.14.4 and 10.17.4, 154-156.
3. Murray, JD (2001) *Mathematical biology I: An introduction*. Springer-Verlag, 510.
